# Genotyping and characterization of prophage patterns in *clinical isolates of Staphylococcus aureus*

**DOI:** 10.1186/s13104-019-4711-4

**Published:** 2019-10-21

**Authors:** Mahya Dini, Leili Shokoohizadeh, Farid Aziz Jalilian, Abbas Moradi, Mohammad Reza Arabestani

**Affiliations:** 1 Department of Microbiology, Faculty of Medicine, School of Medicine, University of Hamadan, Hamadan, Iran; 2 Department of Virology, School of Medicine, University of Hamadan, Hamadan, Iran; 3 Department of Community Medicine, Hamadan University of Medical Sciences, Hamadan, Iran; 4 Nutritious Research Center, Hamadan University of Medical Sciences, Hamadan, Iran

**Keywords:** Methicillin-resistant *Staphylococcus aureus*, Virulence factors, Prophage patterns, rep-PCR

## Abstract

**Objective:**

*Staphylococcus aureus* is considered an important pathogen with a variety of virulence factors in communities and hospitals all around the world. Prophage typing is a practical technique for categorizing this bacterium. In this study, we focused on the detection of prophage patterns in methicillin-resistant *S. aureus* (MRSA) and methicillin-sensitive *S. aureus* (MSSA) strains based on their virulence factors, antimicrobial resistance patterns, and molecular typing by rep-PCR.

**Results:**

Out of 126 *S. aureus* isolates, 45 (35.7%) were identified as MRSA. In total, 17 different prophage types were detected and 112 strains out of 126 strains contained at least one prophage. There was a statistically significant relationship between *hld*, *hlg*, *eta* and SGA, SGA, and SGFb, respectively. The results of the rep-PCR analysis revealed 14 different patterns among the MRSA and MSSA isolates. In conclusion, the presence of different prophage-encoded virulence factors and antibiotic-resistant genes among MRSA strains enables them to produce a broad range of diseases. Thus, diverse MRSA strains which have these prophages can be considered as a potential threat to the patient’s health in either the hospital or the community.

## Introduction

*Staphylococcus aureus* is a gram-positive coccus and is considered a normal flora or an opportunistic pathogen which can cause community-acquired (CA) and hospital-acquired (HA) infections. *S. aureus* can cause a different range of diseases from soft tissue and skin infections to severe life-threatening infections such as toxic shock syndrome (types 1 and 2)[1, 2]. The capability of producing different virulence factors is the main reason for the high pathogenicity of *S. aureus*. In addition to its high pathogenicity, this bacterium also has high adaptive powers against environmental changes [3, 4]. Bacteriophages can convert non-pathogenic strains to pathogenic strains through the horizontal transduction of the virulence gene [5].

The production of virulence factors is the result of the phenotypic changes caused by lysogenic conversion which plays an important role in the biology of these species [6–11]. *S. aureus* phages that are involved in human diseases are classified into six categories: 3 SGA, 11 SGB, 77 SGF (with the two subgroups SGFa and SGFb) and 187 SGL which belong to the siphoviridae family, while SGD or the Twort-like phage belongs to the myoviridae family [2].

In recent years, in epidemiological studies and in assessing the genetic linkages of bacteria, common methods of bacterial typing have been replaced by molecular methods [12]. The rep-PCR technique is an alternative technique for producing fingerprint directly and without using endonuclease enzymes. Staphylococcal bacteriophages are widely used in the typing of Staphylococcus strains associated with human diseases through the rep-PCR technique [13–15]. Given that there are few studies about investigating the relationship between different prophage patterns, virulence factors, antibiotic resistance, SCC*mec*, and rep types in Iran and some other countries, the current study focuses on this field.

## Main text

### Methods

#### Sampling and isolation of *S. aureus*

A total of 126 samples isolated in a previous study by Tahmasebi et al. were used [16]. This study was approved by the ethics committee of Hamadan University of Medical Sciences (No: IRUMSHA. REC. 1397.193).

#### Antibiotic susceptibility testing

For all the *S. aureus* isolates, antibiotic susceptibility testing was done using Kirby–Bauer disk diffusion method according to Clinical and Laboratory Standard Institute (2016) guidelines. The antibiotic disks (Mast, UK) used in this study included cefoxitin (30 μg), tetracycline (10 μg), penicillin (10 u), erythromycin (15 μg), vancomycin (30 μg), cefazolin (10 μg), and ciprofloxacin (5 μg). Methicillin–resistance was detected using a cefoxitin disk and a cefoxitin E-test (Italy, Liofilchem). *S. aureus* ATCC43300 and *S. aureus* ATCC25923 were used as positive and negative controls, respectively.

#### DNA extraction

The total genomic DNAs of the isolates were extracted by the boiling method. The quality of the extracted DNAs was assessed using a Nanodrop device (A & E Lab Nano-200, UK).

#### Detection of virulence factors

Hemolysin genes (*hla*, *hlb*, *hlc*, and *hld*), exfoliative toxins (*et*A, *et*B, and *et*d), Panton–Valentine leukocidin (PVL) genes (*pvl*1, *pvl*2, and *pvl*3), toxic shock syndrome toxin (tsst) and *S. aureus* enterotoxin (seA) genes were identified using the PCR assay as described in our previous studies [16, 17].

#### Detecting the prophages of *S. aureus*

Multiplex PCR assay was used for identifying SGA, SGB, SGD, SGL, and SGF (and its two subgroups SGFa and SGFb) genes by their particular primers. The primers used for *S. aureus* bacteriophages are given in Additional file 1: Table S1. The PCR protocol used was based on the study of Pantuck et al. [1].

#### Detection of SCC*mec* types

SCC*mec* types I to V were detected using a multiplex-PCR assay reported previously [9].

#### Sequencing

One sample of each prophage PCR product (amplicon) was sequenced by Bioneer Co. (South Korea) and the data were analyzed using the Chromas software.

#### Rep-PCR

Rep-PCR was performed on 55 isolates chosen from the MRSA and MSSA strains based on their prophage profiles using the primers indicated in (Additional file 1: Table S1).

#### Analysis of the rep-PCR results

The rep-PCR band patterns were compared and clustered by Dice and unweighted paired group (UPGMA) methods using an online service (the inslico.ehu.es database), respectively.

#### Statistical analysis

The data were analyzed using SPSS software version 16 (Chicago, SPSS Inc., IBM, USA). Descriptive statistical methods were used to determine the frequency, percentage, and mean, while Fisher’s test was used to compare the qualitative results. P ≤ 0.05 was considered as statistically significant.

### Results

#### Prevalence of MRSA strains

Among 126 tested *S. aureus* isolates, 45 strains (35.7%) showed resistance to cefoxitin and were identified as MRSA isolates. All the MRSA isolates carried the *mec*A gene.

#### *SCCmec* typing

The results of the SCC*mec* typing of MRSA strains revealed that these strains were positive for SCC*mec* types I (4%), II (12.7%), III (9.5%) and were classified as hospital-acquired MRSA strains. Furthermore, (15.9%), (2.4%), (4%), and (4%) of the MRSA strains which harbored SCC*mec* types IVa, IVb, IVc, and IVd, respectively, were considered as community-acquired MRSA (Table 1).

**Table 1:** Prevalence of antibiotic resistance genes among the *S. aureus* isolates

	Primer sequence 5′ → 3′	PCR product length (bp)	Primer position	References
SGA1 SGA2	TATCAGGCGAGAATTAAGGG CTTTGACATGACATCCGCTTGAC	744	1409–1428 2152–2130	[1]
SGB1 SGB2	ACTTATCCAGGTGGYGTTATTG TGTATTTAATTTCGCCGTTAGTG	405	1639–1660 2043–2021	[1]
SGF1 SGF2	CGATGGACGGCTACACAGA TTGTTCAGAAACTTCCCAACCTG	155	515–553 669–647	[1]
SGFa1 SGFa2	TACGGGAAAATATTCGGAAG ATAATCCGCACCTCATTCCT	548	2487–2506 3034–3015	[1]
SGFb1 SGFb2	AGACACATTAAGTCGCACGATAG TCTTCTCTGGCACGGTCTCTT	147	20013–20035 20159–20139	[1]
SGL1 SGL2	GCTTAAAACAGTAACGGTGACAGTG TGCTACATCATCAAGAACACCTGG	548	2263–2287 2911–2888	[1]
SGD1 SGD2	TGGGCTTCATTCTACGGTGA GTAATTTAATGAATCCACGAGAT	331	781–800 1111–1089	[1]
REP1 REP 2	IIIICGICGICATCIGGC ICGICTTATCIGGCCTAC	Variable		[18]

#### Prevalence of virulence factors

The prevalence of the virulence factors is given in Additional file 2: Figure S1. The *hla* gene was the prevalent virulence factor (12.7%) and *pvl*1, *pvl*2, and *sea* showed the least (7.9%) distribution among the *S. aureus* isolates.

#### Prevalence of prophages

The results of this study showed that out of 126 strains, 112 contained at least one prophage (Additional file 3: Figure S2). SGB (88%) was the most prevalent prophage, while SGL and SGA were the least prevalent prophages (3.2%). All of the phage types were detected in the MRSA and MSSA strains, except for SGL which was detected only in the MRSA strains. In total, 17 different prophage types were detected among the *S. aureus* isolates in this study (Table 2).

**Table 2:** Prophage patterns of *S. aureus* isolates

Resistance gene	Negative	Positive
*mecA*	81 (64/3)	45 (35/7)
*mecR*	114 (90/5)	12 (9/5)
*mecI*	121 (96)	5 (4)
*ccra.a4*	106 (84/1)	20 (15/9)
*ccra.b3*	112 (88/9)	14 (11/1)
*ccra.b2*	103 (81/7)	23 (18/3)
*ccra.b1*	110 (92/1)	16 (7/9)
*ccar2.b*	121 (96)	5 (4)
*SCCmecI*	116 (92/1)	10 (7/9)
*SCCmecII*	110 (87/3)	16 (12/7)
*SCCmecIII*	114 (90/5)	12 (9/5)
*SCCmecIVa*	106 (84/1)	20 (15/9)
*SCCmecIVb*	123 (97/6)	3 (2/4)
*SCCMECIVc*	121 (96)	5 (4)
*SCCmecIVd*	121 (96)	5 (4)
*ccrC*	117 (92/9)	9 (7/1)
*ermA*	121 (96)	5 (4)
*ermB*	117 (92/9)	9 (7/1)
*ermC*	112 (88/9)	14 (11/1)
*BlaZ*	0 (0)	126 (100)

#### The relationship between prophages and virulence factors

By using Fisher’s test, it was noticed that the P. values of *hld*/SGA, *hlg*/SGA, and *eta*/SGFb were 0.05, 0.04, and 0.04, respectively. This shows that there is a statistically significant relationship between these virulence factors and their related prophages.

#### The relationship between prophages and SCC*mec* types

The comparison of the antibiotic resistance genes and the presence of prophages showed that there was a statistically significant relationship between: SGB and *mec*A; SGFa and *mec*A, *mec*R, and mecC; SGFb and *mec*A and SCC*mec* IV; SGL and *mec*R and SCC*mec* IVa; and SGF and *mec*A (Table 3).

**Table 3:** The relationship between prophages and antimicrobial resistance genes based on the P values

Phage pattern	Pahe type							No (%)
	SGB	SGD	SGFa	SGFb	SGA	SGF	SGL	
Pattern 1	−	−	+	+	−	−	−	12
Pattern 2	−	+	+	−	−	+	−	3
Pattern 3	−	+	+	−	−	−	−	7
Pattern 4	+	−	+	+	−	−	−	2
Patten 5	−	−	+	+	−	+	−	9
Pattern 6	−	−	+	−	−	+	−	48
Pattern 7	−	−	−	+	−	+	−	13
Pattern 8	−	−	+	−	+	+	−	3
Pattern 9	+	−	+	−	−	+	−	14
Pattern 10	+	−	−	−	−	−	+	2
Pattern 11	+	−	−	+	−	+	−	6
Pattern 12	−	+	−	−	−	+	+	1
Pattern 13	−	+	+	−	−	−	−	1
Pattern 14	−	+	−	−	−	−	+	1
Pattern 15	+	+	−	−	−	−	+	1
Pattern 16	−	+	−	−	−	−	−	2
Pattern 17	−	−	−	−	−	−	+	1

#### The results of rep-PCR

The analysis of genetic linkage among the isolates using rep-PCR showed a similarity of 50–100% among the *S. aureus* isolates (Additional file 4: Figure S3). Genetic diversity was established among the *S. aureus* isolates by detecting 14 different rep-PCR fingerprints with the similarity cutoff of ≥ 95%. Fourteen different rep-PCR profiles, including seven common types and seven unique types, were identified.

### Discussion

In the present study, the frequency of resistance to methicillin was 35.7%. Most studies in Iran have reported the prevalence of MRSA from 43 to 56% [19, 20]. However, the rate of MRSA in our study is lower than those of some studies in Iran and other countries.

Our findings revealed that all the prophage types (SGA, SGB, SGD, SGF, SGFa, SGFb, and SGL) were detected among the MRSA and MSSA strains and the SGB prophage type was the predominant one. However, SGF prophage has been the predominant prophage type in previous reports from 2012 to 2018 in Iran [2, 19–22]. In the current study, the SGD prophage was also detected among the *S. aureus* strains. Rahimi et al. reported four different prophage patterns and six different prophage types among the MRSA strains isolated from Tehran hospitals in Iran [20, 23]. However, in our study, seventeen different prophage patterns and seven different prophage types were detected. In contrast to a previous study in Iran in which the prophage pattern SGB/SGF/GFa/SGFb (81%) was reported as the dominant pattern, in the current study, SGF/SGFa was identified as the dominant pattern in 38% of the isolates [22].

Another difference between the findings of the present research and those of other researches in Iran is that SGL was found among the studied strains of this study while it was not reported in previous studies.

According to the results of the current study, there was not a significant relationship between resistance to methicillin and prophage types. All prophage types were detected among both MRSA and MSSA strains except for SGL which was detected only among the MRSA isolates. In the present study, the *hla* gene detected in 12% of the strains was the predominant hemolysin gene. The *hlb* gene detected in 11% of the *S. aureus* strains was the second dominant hemolysin gene, though it was the predominant hemolysin gene and was detected in 100% of the MRSA strains in a study by Rahimi et al. in Tehran hospitals in 2018 [23]. According to the results of the current study, both MRSA and MSSA strains had the *hla* gene, however, the prevalence of this gene was significantly more in the MRSA strains which proves the relationship between this virulence factor and methicillin resistance in *S. aureus*. On the other hand, there was a statistically significant relationship between SGA and *hld* which is another hemolysin gene.

Another statistically significant relationship between prophages and virulence factors was the one between *eta* and SGFb. In contrast to Rahimi et al. [23] who showed that there was a relationship between *eta* and SGB, the authors of the current study did not find the same result. In the current study, the frequency of *sea*, *hemolysin*, *and tst* genes was lower than those of other studies in Iran and other countries [19, 24—26]. One of the reasons for the low abundance of these genes may be the low number of samples tested in this study compared to those of similar studies. However, the frequency of *pvl* genes was a little higher than those of other studies in Iran. PVL is one of the most important virulence factors and a marker for SCC*mec* type II which is associated with SGA phages [2, 27]. In the current study, *pvl* genes were found among strains with SCCmec (types I, II, III, IV), and SGF, SGD, and SGB phages. This shows the community origins of these strains because the presence of the *pvl* gene is only limited to CA-MRSA strains. According to the PCR results, 28 out of 126 (22.22%) *S. aureus* isolates were *pvl* carriers. In 2013, Abimanyu et al. in India reported that 10 out of 25 (40%) MRSA isolates were positive for *pvl* genes [28] which was higher than the results of our study. In another study in Iran, Ohadian Moghadam et al. showed that 14.3% of the 56 isolated *S. aureus* collected from Isfahan were PVL-positive [29].

In the current study, SCC*mec* I, II, III were detected among the HA-MRSA strains and SCC*mec* IVa, IVb, IVc, IVd were found among the CA-MRSA strains. Consistent with previous studies in Iran and in most Asian countries, SCC*mec* type III was found as the dominant SCC*mec* type among the HA-MRSA isolates [19, 30, 31]. In the current study, SCC*mec* IVa showed a higher frequency among the *SCCmec* types. This type was reported by Edselve et al. in Denmark. In the current study, SCC*mec* type V was detected in none of the isolates.

In the current study, *S. aureus* isolates were categorized by SCC*mec* typing, phage typing, and rep-PCR. In the study of Sommerhalter et al. it has been shown that the performance of rep-PCR typing is comparable to those of other typing methods. The analysis of the linkage among the isolates by rep-PCR showed a genetic diversity among the *S. aureus* isolates (14 different rep-PCR profiles including seven common types and seven unique types). The common types including A and G harbored 10 and 14 isolates, respectively. Isolates in the same rep profiles showed different phage patterns, virulence genes, SCC*mec* types, and *pvl* genes. Therefore, it can be concluded that there is no significant relationship between the rep patterns and the phage profiles, virulence genes, and SCC*mec* types. The results of studies from Iran and other countries indicated diversity among MRSA isolates from hospitals and the community. In a study by Manafi et al. in Iran [32], six main clusters of *S. aureus* were detected by rep-PCR and no significant relationship was observed between PVL-positive *S. aureus* and rep-PCR patterns. This is in line with our findings.

### Conclusion

Our findings illustrated the high diversity of different prophage types, rep types, and SCC*mec* types in both MRSA and MSSA strains. According to the results of the rep-PCR technique, we also face diverse *S. aureus* isolates in hospitals in Hamedan and their circulation from the community to the hospitals and vice versa.

## Limitation

The results of this study had some similarities and discrepancies with those of other studies. It is necessary to conduct studies with more samples in different areas and to use robust typing methods to obtain better results for better interpretations.

## Supplementary information

**Additional file 1: Table S1.** Primer sequences used in the prophage typing and rep-PCR in the *S. aureus* isolates.

**Additional file 2: Figure S1.** Prevalence of virulence factors among 126 clinical isolates of *S. aureus.*

**Additional file 3: Figure S2.** Prevalence of prophage types among 126 clinical isolates of *S. aureus*

**Additional file 4: Figure S3.** The dendrogram of rep-PCR analysis for 55 clinical isolates of *S. aureus*. The common types are marked.

## Data Availability

The supporting data files include Additional file 1: Table S1 and Additional file 2: Figures S1, Additional file 3: Figures S2, Additional file 4: Figures S3.

## Abbreviations

MRSA: Methicillin-resistant *Staphylococcus aureus*
MSSA: Methicillin-sensitive *Staphylococcus aureus*
HGT: horizontal gene transfer
PVL: Panton–Valentine leukocidin
*sea*: enterotoxin gene
*tst*: toxic shock toxin
