# Supplementary material for: Genotyping and characterization of prophage patterns in clinical isolates of Staphylococcus aureus
Source: BMC Res Notes. 2019 Oct 21;12:669. doi: 10.1186/s13104-019-4711-4 (PMC6805666; doi:10.1186/s13104-019-4711-4)
Supplement: Supplementary file 1 — Additional file 1: Table S1. Primer sequences used in the prophage typing and rep-PCR in the S. aureus isolates. [file 13104_2019_4711_MOESM1_ESM.docx]

**Table S1:**

| reference | Primer position | PCR product length(bp) | Primer sequence5'🡪3' | |
| --- | --- | --- | --- | --- |
| (1) | 1409-1428  2152-2130 | 744 | TATCAGGCGAGAATTAAGGG  CTTTGACATGACATCCGCTTGAC | SGA1 SGA2 |
| (1) | 1639-1660  2043-2021 | 405 | ACTTATCCAGGTGGYGTTATTG  TGTATTTAATTTCGCCGTTAGTG | SGB1 SGB2 |
| (1) | 515-553  669-647 | 155 | CGATGGACGGCTACACAGA  TTGTTCAGAAACTTCCCAACCTG | SGF1 SGF2 |
| (1) | 2487-2506  3034-3015 | 548 | TACGGGAAAATATTCGGAAG  ATAATCCGCACCTCATTCCT | SGFa1 SGFa2 |
| (1) | 20013-20035  20159-20139 | 147 | AGACACATTAAGTCGCACGATAG  TCTTCTCTGGCACGGTCTCTT | SGFb1 SGFb2 |
| (1) | 2263-2287  2911-2888 | 548 | GCTTAAAACAGTAACGGTGACAGTG  TGCTACATCATCAAGAACACCTGG | SGL1  SGL2 |
| (1) | 781-800  1111-1089 | 331 | TGGGCTTCATTCTACGGTGA  GTAATTTAATGAATCCACGAGAT | SGD1  SGD2 |
| (18) |  | variable | IIIICGICGICATCIGGC ICGICTTATCIGGCCTAC | REP1 REP 2 |
