# Supplementary figures and images for: Genotyping and characterization of prophage patterns in clinical isolates of Staphylococcus aureus
Source: BMC Res Notes. 2019 Oct 21;12:669. doi: 10.1186/s13104-019-4711-4 (PMC6805666; doi:10.1186/s13104-019-4711-4)

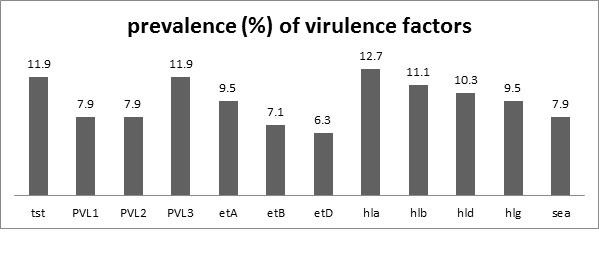

Supplement: Supplementary file 2 — Additional file 2: Figure S1. Prevalence of virulence factors among 126 clinical isolates of S. aureus. [file 13104_2019_4711_MOESM2_ESM.jpg]

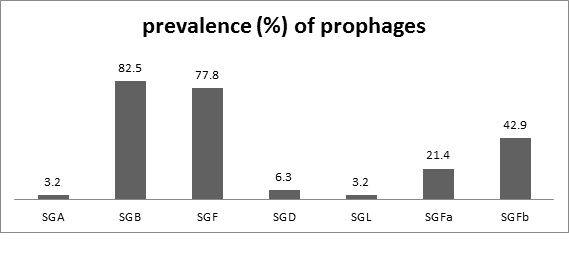

Supplement: Supplementary file 3 — Additional file 3: Figure S2. Prevalence of prophage types among 126 clinical isolates of S. aureus [file 13104_2019_4711_MOESM3_ESM.jpg]

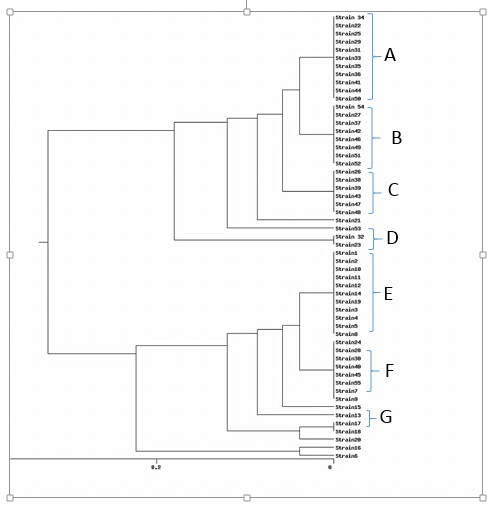

Supplement: Supplementary file 4 — Additional file 4: Figure S3. The dendrogram of rep-PCR analysis for 55 clinical isolates of S. aureus. The common types are marked. [file 13104_2019_4711_MOESM4_ESM.jpg]
